# Green silver nanoparticles of *Phyllanthus amarus*: as an antibacterial agent against multi drug resistant clinical isolates of *Pseudomonas aeruginosa*

**DOI:** 10.1186/s12951-014-0040-x

**Published:** 2014-10-01

**Authors:** Khushboo Singh, Manju Panghal, Sangeeta Kadyan, Uma Chaudhary, Jaya Parkash Yadav

**Affiliations:** Department of Genetics, M.D. University, Rohtak, 124001 Haryana India; Department of Microbiology, Pt. B.D.S Post Graduate Institute of Medical Sciences Rohtak, Rohtak, 124001 Haryana India

**Keywords:** *Pseudomonas aeruginosa*, Burn isolates, MDR, *Phyllanthus amarus*, Silver nanoparticles, Antibacterial

## Abstract

**Background:**

*Pseudomonas aeruginosa* infection is a leading cause of morbidity and mortality in burn and immune-compromised patients. In recent studies, researchers have drawn their attention towards ecofriendly synthesis of nanoparticles and their activity against multidrug resistant microbes. In this study, silver nanoparticles were synthesized from aqueous extract of *Phyllanthus amarus*. The synthesized nanoparticles were explored as a potent source of nanomedicine against MDR burn isolates of *P. aeruginosa*.

**Results:**

Silver nanoparticles were successfully synthesized using *P. amarus* extract and the nature of synthesized nanoparticles was analyzed by UV-Vis spectroscopy, transmission electron microscopy, energy dispersive X-ray spectroscopy, dynamic light scattering, zeta potential, X- ray diffraction and fourier transform infra-red spectroscopy. The average size of synthesized nanoparticles was 15.7, 24 ± 8 and 29.78 nm by XRD, TEM and DLS respectively. The antibacterial activity of AgNPs was investigated against fifteen MDR strains of *P. aeruginosa* tested at different concentration. The zone of inhibition was measured in the range of 10 ± 0.53 to 21 ± 0.11mm with silver nanoparticles concentration of 12.5 to 100 μg/ml. The zone of inhibition increased with increase in the concentration of silver nanoparticles. The MIC values of synthesized silver nanoparticles were found in the range of 6.25 to12.5 μg/ml. The MIC values are comparable to the standard antibiotics.

**Conclusion:**

The present study suggests that silver nanoparticles from *P. amarus* extract exhibited excellent antibacterial potential against multidrug resistant strains of *P. aeruginosa* from burn patients and gives insight of their potential applicability as an alternative antibacterial in the health care system to reduce the burden of multidrug resistance.

**Electronic supplementary material:**

The online version of this article (doi:10.1186/s12951-014-0040-x) contains supplementary material, which is available to authorized users.

## Background

*Pseudomonas aeruginosa*, a gram negative bacterium, is the leading cause of morbidity and mortality in burn patients as they are more susceptible to infections because of immune-suppression and loss of cutaneous coverage [1]. Since *P. aeruginosa* has innate potential to develop resistance, virtually to any antibiotics to which it is exposed, due to the presence of multiple resistance mechanisms and it becomes a multidrug resistant (MDR) strain. Infections caused by MDR *P. aeruginosa* are often severe; life threatening and these strains have frequently been reported as the cause of nosocomial infections. These MDR have been emerged as a major problem in burn units as burn injury disrupts both the normal skin barrier and many of systemic host defence mechanism which make burn patients the ideal hosts for opportunistic infections [2]. The importance to prevent these infections has been recognized since its inception thus it becomes difficult to treat the infection caused by *P. aeruginosa* MDR strains due to their narrow range of susceptibility to antimicrobial agents. Therefore, currently, researchers started to develop alternative therapies to aid patients to recover from the infections. In future, these alternatives may be useful in treating not only burn infections but other antibiotic resistant infections as well.

Nanotechnology provides a good platform to modify and develop the important properties of silver metal in the form of nanoparticles having promising applications as an antibacterial agent [3,4]. Silver nanoparticles have high surface area to volume ratio and the unique chemical, physical properties [5,6]. Nowadays, they have been widely used as an effective bactericidal agent against broad spectrum of bacteria, including antibiotic resistant strains [7]. Hence, researchers are shifting towards nanoparticles in general and silver nanoparticles (AgNPs) in particular to solve the problem of emergence of MDR bacteria [8]. Also the development of biological approach for the synthesis of nanoparticles is evolving in to an important branch of nanotechnology [9,10]. The biological method has advancement over chemical and physical method as it is cost effective and ecofriendly [11,12]. *Phyllanthus amarus* is an important plant of Indian Ayurvedic system of medicine, belongs to the family Euphorbiaceae. It is a small herb well known for its medicinal properties and has been used worldwide [13].

This study aims to explore the efficacy of synthesized silver nanoparticles from *P. amarus* as a potent source of nanomedicine against MDR burn isolates of *P. aeruginosa* and establish the therapeutic antibacterial potential of plant with nanotechnology; thereby justify the folklore claim of the plant used in the traditional system of Indian medicine.

## Results

### Synthesis of AgNPs

The AgNPs were successfully synthesized using aqueous plant extract of *P. amarus* by mixing with silver nitrate solution (1mM). The colour changes from pale yellow to dark brown (Additional file 1: Figure S1). This was observed due to the reduction of Ag^+^ and it indicates the formation of AgNPs.

### Characterization of Ag nanoparticles

**Figure 1:**
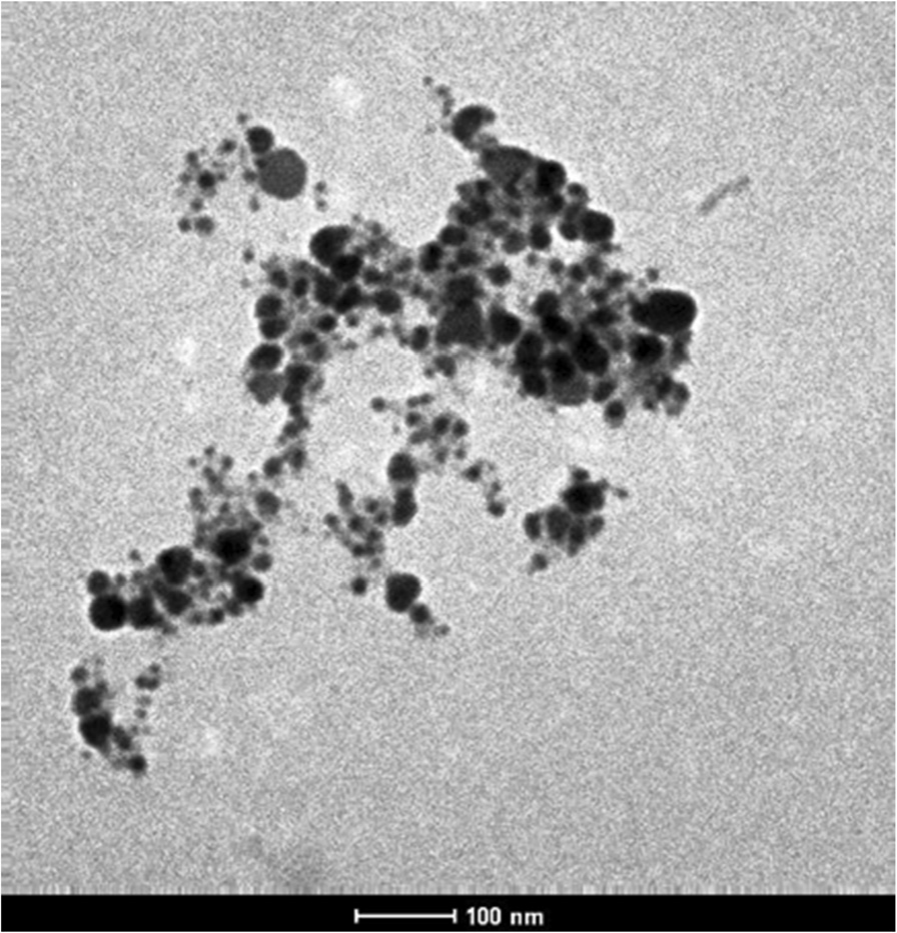
**Image of TEM of silver nanoparticles of** ***P. amarus.*** Figure showing picture of transmission electron microscopy of silver nanoparticles of *P. amarus.*

**Figure 2:**
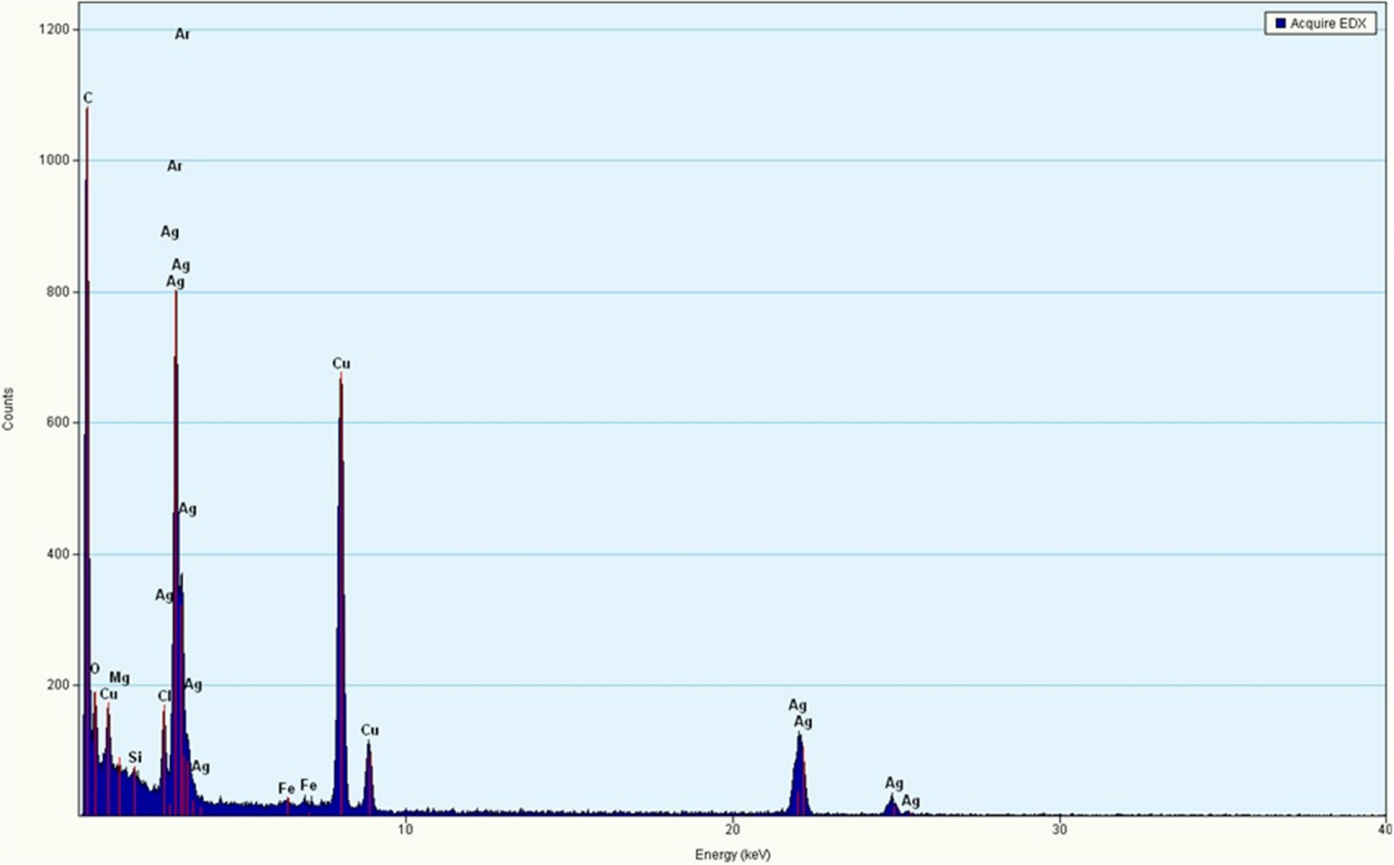
**Image of EDX of silver nanoparticles of** ***P. amarus.*** Figure showing picture of energy dispersive x-ray spectroscopy of silver nanoparticles of *P. amarus.*

The synthesis of AgNPs was confirmed by UV-VIS spectrophotometer (Shimadzu). The UV-VIS absorption spectra of the AgNPs were monitored in a range of 300-800 nm. A strong peak specific for the synthesis of silver nanoparticles was obtained at 420-430 nm. Additional file 2: Figure S2 shows the absorption spectra of AgNPs synthesized by *P. amarus*. The TEM results (Figure 1) showed that all synthesized AgNPs were spherical in shape with 24 ± 8 nm size and found to be well dispersed in aqueous medium. EDX characterization has shown absorption of strong silver signal along with other elements, which may be originated from the biomolecules that are bound to the surface of silver nanoparticles. EDX performed by energy and intensity distributions of X-ray signals generated by focused electron beam on a specimen. From EDX spectra, showed in Figure 2, it is clear that silver nanoparticles reduced by *P. amarus.*

**Figure 3:**
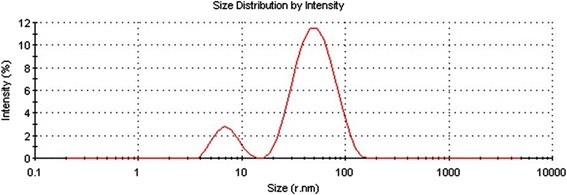
**DLS graph of silver nanoparticles of** ***P. amarus.*** Figure showing graph of dynamic light scattering of silver nanoparticles of *P. amarus.*

**Figure 4:**
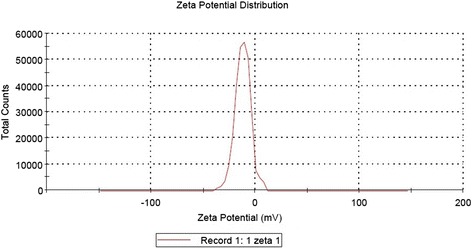
**Zeta potential graph of silver nanoparticles of** ***P. amarus.*** Figure showing graph of zeta potential of silver nanoparticles of *P. amarus.*

**Figure 5:**
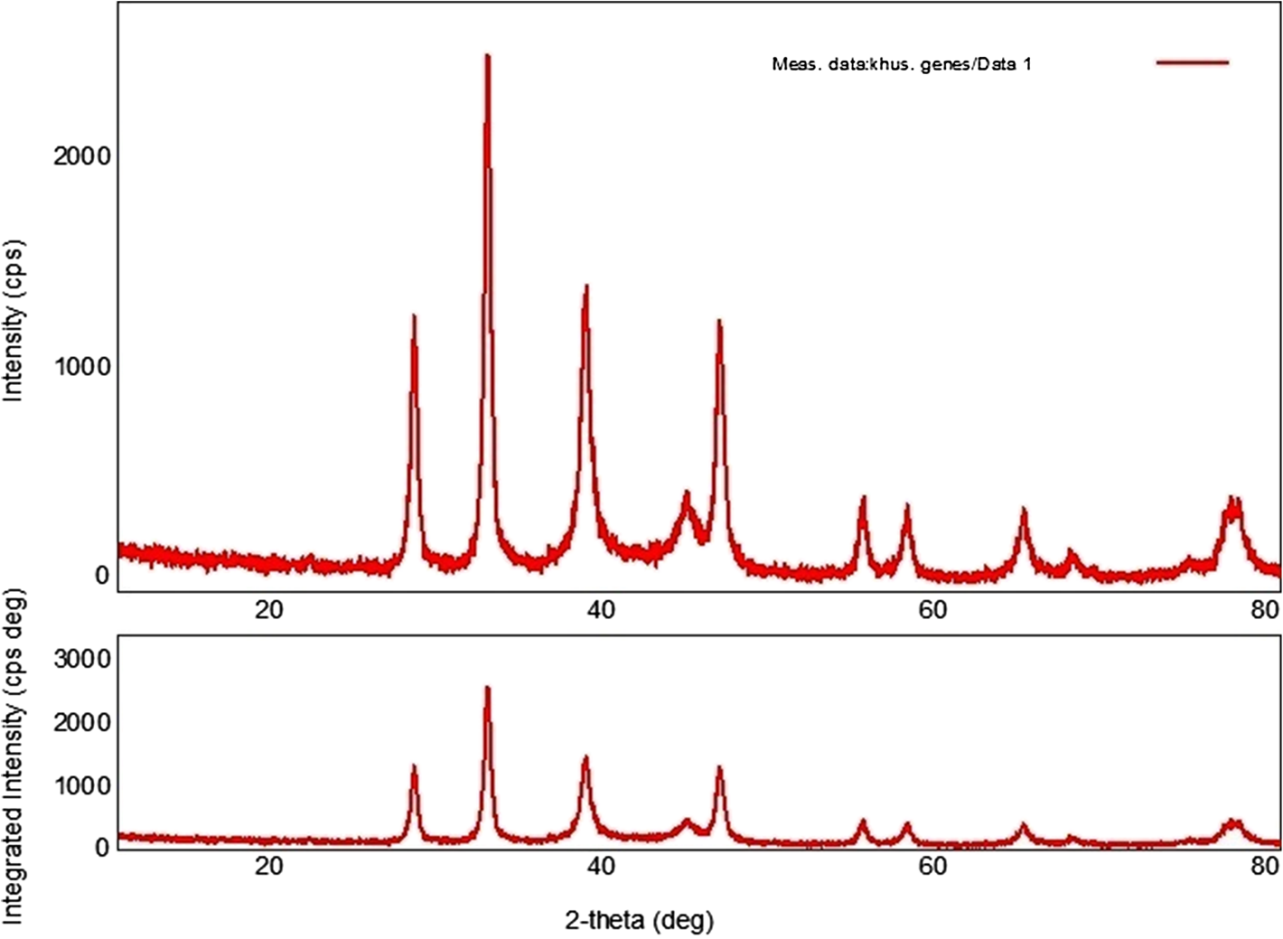
**XRD of silver nanoparticles of** ***P. amarus.*** Figure showing X-Ray Diffraction pattern of silver nanoparticles of *P. amarus.*

**Figure 6:**
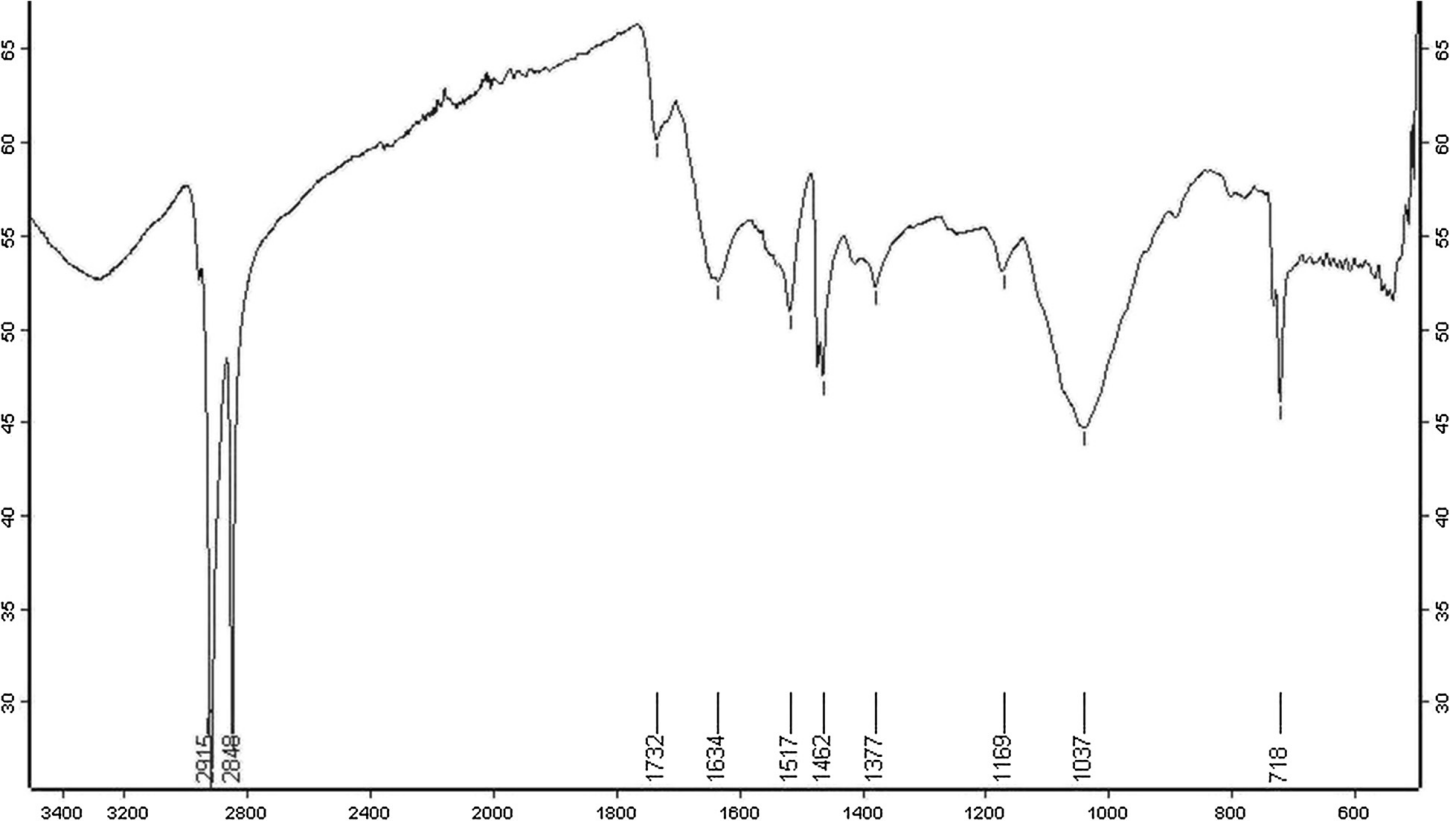
**FTIR of silver nanoparticles of** ***P. amarus.*** Figure showing Fourier Transform Infra-Red spectroscopy of silver nanoparticles of *P. amarus.*

Dynamic light scattering (DLS) technique and zeta potential has been used to determine the size of particles and measure the potential stability of the particles in the colloidal suspension respectively. Figure 3 and Figure 4 have shown the DLS and zeta potential graph of AgNPs of *P. amarus* with an average size of 29.78 nm and the particles carry a charge of -11.9 mV respectively. Silver nanoparticles generally carry a negative charge. All silver nanoparticles synthesized from *P. amarus* showed negative charge and were stable at room temperature. The particle size and nature of AgNPs was determined by XRD. The mean particle diameter of AgNPs was calculated using the Debye-Scherrer’s equation. An average size of the silver nanoparticles synthesized by *P. amarus* was 15.7 nm (Figure 5 and Table 1). The FT-IR spectrum of AgNPs from *P. amarus* showed the characteristics absorbance bands (Figure 6) due to aldehydic C–H stretch (2,915 and 2,848 cm^-1^), C-O stretch (1,634 cm^-1^), N-H (1517 cm^-1),^ 1462 cm^-1^ N-O stretch (1,377 cm^-1^) and C-O stretch (dialkyl) (1,169 cm^-1^), C-N (1,037 cm^-1^), C-H stretch (718 cm^-1^).

**Table 1:** **Size of AgNPs of** ***P. amarus*** **by using Debye-Scherrer’s equation**

**S. No.**	**2-theta(deg)**	**D (ang.)**	**FWHM(deg)**	**Int. I(cps deg)**	**Int. W(deg)**	**Size(nm)**
1	27.797	3.206	0.464	488.7	0.651	18.4
2	32.210	2.776	0.450	1008.65	0.622	19.1
3	38.094	2.306	0.647	845.25	0.992	13.5
4	44.195	2.047	1.204	224.79	1.535	7.43
5	46.250	1.961	0.576	492.67	0.702	15.6
6	54.750	1.675	0.532	152.66	0.722	17.5
7	57.450	1.602	0.610	148.96	0.825	15.4
8	64.485	1.443	0.367	171.27	0.758	26.6
9	77.068	1.236	1.285	258.17	1.368	8.25

### Antibacterial assay

**Figure 7:**
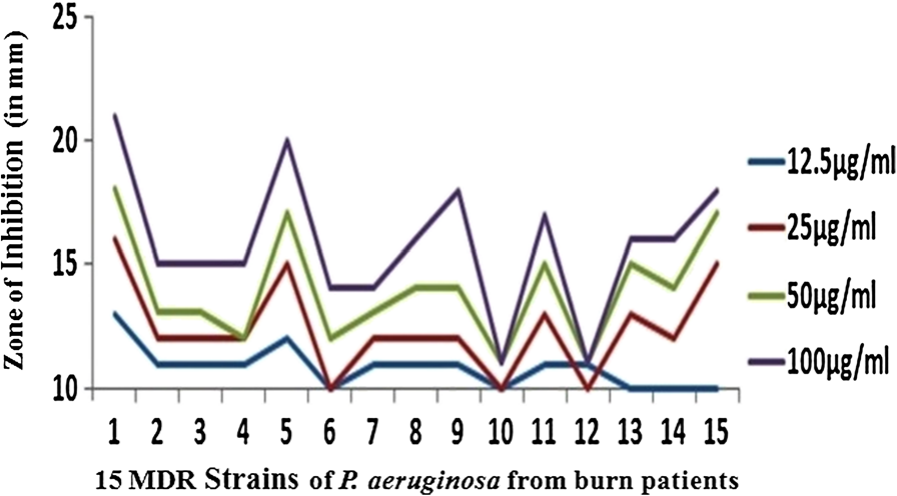
**Graph showing antibacterial activity of silver nanoparticles of** ***P. amarus.*** Figure showing graph of antibacterial activity of silver nanoparticles of *P. amarus* at different concentration *against* 15 MDR strains of *P. aeruginosa* from burn patients.

**Figure 8:**
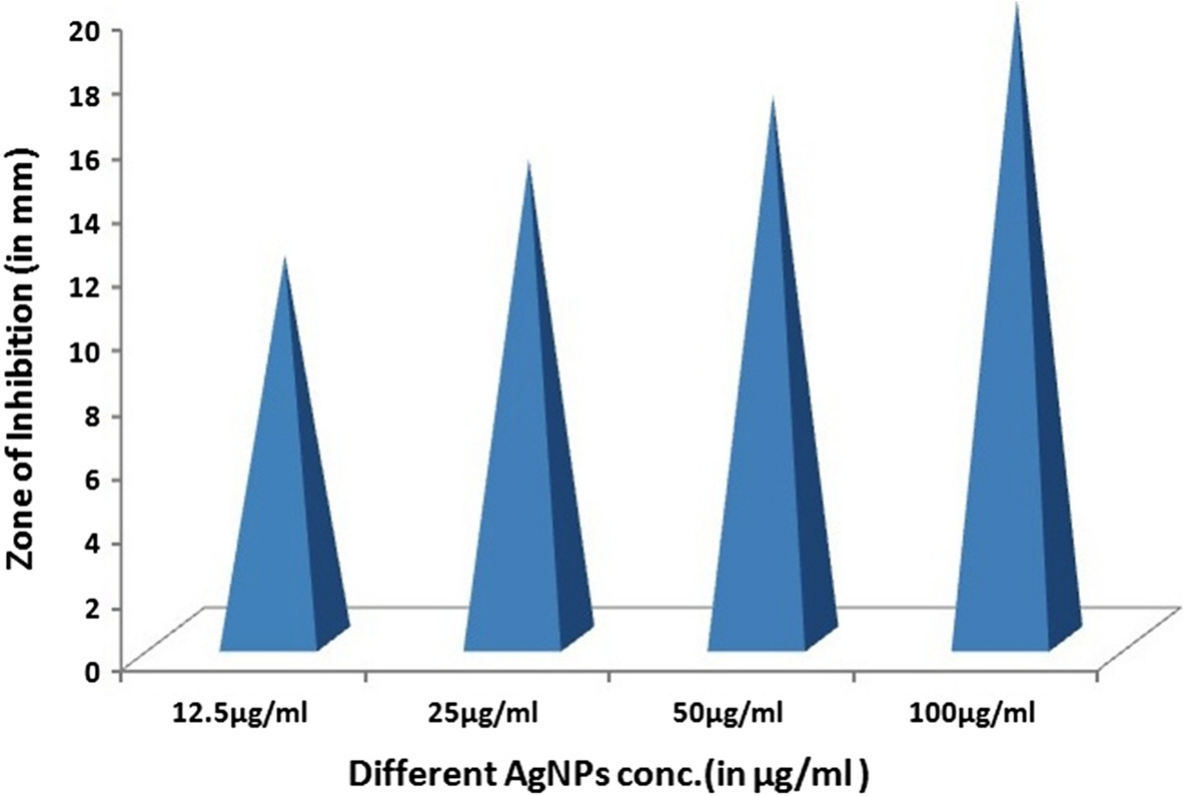
**Graph showing antibacterial activity of silver nanoparticles of** ***P. amarus against*** **representative MDR strain** ***.*** Figure showing graph of antibacterial activity of silver nanoparticles of *P. amarus* at different concentration *against* MDR strain 1 of *P. aeruginosa* from burn patients.

The 15 multidrug resistant strains of *P. aeruginosa* isolated from burn patients tested at various concentrations of AgNPs i.e. 12.5, 25, 50 and 100 μg/ml to determine the antibacterial effect by agar well diffusion method. The AgNPs showed (Additional file 3: Figure S3) antimicrobial activity against all the tested pathogens. The antibacterial activity is concentration dependent as it increased with the concentration of AgNPs (Figure 7). The zone of inhibition measured in a range of 10 ± 0.53 to 21 ± 0.11 mm. MDR Strain1 was found (Figure 8) to be most susceptible where zone of inhibition ranged from 13 ± 1 to 21 ± 0.11 mm at AgNPs concentration of 12.5 to 100 μg/ml. MDR strain 10 was least susceptible with 10 ± 0.53 to 13 ± 0.41mm zone of inhibition.

### Minimum Inhibitory Concentration (MIC)

The MIC of AgNPs from *P. amarus* against MDR strains of *P. aeruginosa* was 6.25-12.5 μg/ml. MDR strains 6, 10,12,13,14 and 15 showed the MIC values of 12.5 μg/ml. The remaining nine MDR strains have shown the MIC at 6.25 μg/ml (Table 2) which is lower than standard antibiotic.

**Table 2:** **MIC of silver nanoparticles of** ***P. amarus*** **against MDR strains of** ***P. aeruginosa*** **from burn patients**

**S.No**	**MIC(in μg/ml)**
*P. aeruginosa* MDR Strain 1	6.25
*P. aeruginosa* MDR Strain 2	6.25
*P. aeruginosa* MDR Strain 3	6.25
*P. aeruginosa* MDR Strain 4	6.25
*P. aeruginosa* MDR Strain 5	6.25
*P. aeruginosa* MDR Strain 6	12.5
*P. aeruginosa* MDR Strain 7	6.25
*P. aeruginosa* MDR Strain 8	6.25
*P. aeruginosa* MDR Strain 9	6.25
*P. aeruginosa* MDR Strain 10	12.5
*P. aeruginosa* MDR Strain 11	6.25
*P. aeruginosa* MDR Strain 12	12.5
*P. aeruginosa* MDR Strain 13	12.5
*P. aeruginosa* MDR Strain 14	12.5
*P. aeruginosa* MDR Strain 15	12.5

## Discussion

The biosynthesis of nanoparticles has received considerable attention due to the growing need to develop environmentally benign technologies in material synthesis [14]. The phytochemicals derived from plant products serve as a prototype to develop less toxic and more effective medicines for controlling the growth of microorganisms [15]. These compounds have significant therapeutic application against human pathogens. Numerous studies have been conducted with the extracts of various plants for screening of antimicrobial activity in search of new antimicrobial compounds [16]. *P. amarus* was also reported to have antibacterial efficacy against some drug resistant pathogenic bacterial strains [17]. But there are still limited studies regarding antibacterial activity of AgNPs from this plant. The beauty of the present study is that AgNPs reduced by *P. amarus* were highly effective against MDR burn isolates of *P. aeruginosa* in term of novelty. We synthesized AgNPs from *P. amarus*, which is easily available in rainy season, safe, non-toxic and have a variety of secondary metabolites that can help in the reduction of silver ions. The main mechanism considered for the process is plant-assisted reduction due to phytochemicals. The main phytochemicals involved are terpenoids, flavones, ketones, aldehydes, amides, and carboxylic acids. Flavones, organic acids, and quinones are water-soluble phytochemicals that are responsible for the immediate reduction of the ions [18]. Studies have revealed that *P. amarus* contain mainly phyllanthin, hypophyllanthin, phyltertralin and many more other phytochemicals [19]. It was also suggested that the phytochemicals are involved directly in the reduction of the ions and formation of silver nanoparticles [20]. Though the exact mechanism involved in each plant varies as due to the presence of different phytochemicals which are involved in the reduction of the ions leads to the synthesis of AgNPs. A strong peak was obtained at 420-430 nm showing the absorption spectra of AgNPs synthesized by *P. amarus*. Further EDX has shown absorption of strong silver signal along with other elements that are bound to the surface of silver nanoparticles. TEM, XRD, DLS revealed the size and zeta potential contributed towards the stability of AgNPs [21]. FTIR confirms the presence of different functional groups absorb characteristic frequencies of IR radiations [22].

The exact mechanism by which silver nanoparticles employ to cause antimicrobial effect is not clearly known. However, there are various theories suggested about the action of AgNPs on microbes to cause the antimicrobial effect. The AgNPs have ability to anchor to the bacterial cell wall and subsequently penetrate it, thereby causing structural changes in the cell membrane like the permeability of cell membrane and death of the cell. There is formation of ‘pits’ on the cell surface where accumulation of the nanoparticles takes place [23]. The formation of free radicals by AgNPs may be considered to be another mechanism by which the cells die [24,25]. It has also been proposed that there can be release of silver ions by the nanoparticles [26], and these ions can interact with the thiol groups of many vital enzymes and inactivate them [27]. The bacterial cells in contact with silver absorb silver ions, which inhibit several functions in the cell and damage the cells.

In recent years, due to the development of resistant strains, antibiotic resistance also has been increased. MDR *P. aeruginosa* strains from burn patients are causing serious infections and exhibit innate resistance to many antibiotics. These can develop new resistance after exposures to antimicrobial agents. Some antimicrobial agents are extremely irritant and toxic. The studies on drug resistant bacteria in this facet are still limited. Also AgNPs have gained insight as an excellent antimicrobial agent due to its non-toxic effect on human cells in its low concentration and weaker ability to develop resistance towards silver ions [28-30].

The various researchers showed that AgNPs of *P. amarus* were found to be good antibacterial agent. Humberto et al. [31] showed the antibacterial activity of AgNPs against multidrug-resistant *P. aeruginosa*, *E. coli*, *Streptococcus* sp. and *S. pyogens*. Kathireshwari et al. [32] showed the antimicrobial activity against multi drug resistant human pathogens from leaf mediated synthesis of AgNPs using *Phyllanthus niruri*. Durairaj et al. [33] studied the antibacterial activity of purchased AgNPs (size 20-30 nm) against 10 isolates of *P. aeruginosa* comprising of 5 MDR strains with an inhibition zone of 11 mm observed with10 μg dose of the nanoparticles. In our results, AgNPs showed excellent antibacterial activity which is better than our previous study [34], which showed the good antibacterial activity of AgNPs prepared using *T. cordifolia* aqueous extract against *P. aeruginosa* MDR strain from burn patient with maximum concentration of 200 μg/ml. However, there is vital need and much interest in finding ways to formulate new types of safe and cost-effective biocidal materials [22]. Therefore, in this study, we used different plant as biomaterial and evaluated its antibacterial effects. The synthesized AgNPs showed significant antibacterial activity at concentration of 12.5-100 μg/ml against MDR strains of *P. aeruginosa* isolates. The MIC of AgNPs was found to be in a range from 6.25-12.5 μg/ml, almost nine MDR strains have shown the MIC at 6.25 μg/ml which was lower than that of the standard antibiotic (10 μg). As infection of *P. aeruginosa* always remains one of the most challenging concerns in burn units and the synthesized AgNPs of *P. amarus* are highly effective antibacterial agent against these MDR burn isolates.

## Conclusion

In conclusion, we have demonstrated that AgNPs from *P. amarus* exhibit excellent antibacterial potential against MDR *P. aeruginosa* strains isolated from burn patients. Therefore these AgNPs may act as ecofriendly antibacterial agent against these nosocomial strains and can provides a potent alternative nanomedicine in the health care system to reduce the burden of multidrug resistance.

## Material and methods

### Synthesis of silver nanoparticles from plant extract

#### Preparation of the extract

The whole plant of *Phyllanthus amarus* was collected locally from Botanical Garden, M.D. University, Rohtak, Haryana, India. It was thoroughly washed in distilled water, cut into fine pieces. 10g of fresh plant material was boiled into 100 ml sterile distilled water for 10 minutes and filtered through Whatman’s No.1 filter paper. The extract was stored at 4°C for further experiments.

### Synthesis of AgNPs from plant extract

For synthesis of AgNPs, the above plant extract of *P. amarus* was used and 15 ml of this extract was added to 200 ml of aqueous silver nitrate solution (1mM). This solution was kept for 20 minutes at 70°C (in water bath). The plant extract act as reducing as well as stabilizing agent in the solution and leads to the formation of AgNPs.

### Characterization of synthesized AgNPs

The seven different characterization techniques were used for AgNPs. At first, AgNPs were characterized by UV-VIS Spectroscopy using Shimadzu UV-VIS Spectrophotometer. The scanning range for the samples was 300-800 nm. The double distilled water used as a blank reference. To remove any free biomass residue or compound that is not the capping ligand of the nanoparticles, after complete reduction, silver nanoparticles were concentrated by repeated centrifugation (3 times) of the reaction mixture at 15,000rpm for 20 min. The supernatant was replaced by distilled water each time. Thereafter, the purified suspension was freeze dried to obtain dried powder. The shape and size of AgNPs was determined by transmission electron microscopy (TEM). A drop (2 ul) of water dissolved synthesized nanoparticles was placed on a copper grid. The images were obtained with a Tecnai, Twin 200 KV (FEI, Netherlands) at a bias voltage of 200 kV used to analyze samples. The composition of the AgNPs was determined using the Energy Dispersive X-Ray Spectroscopy (EDX) coupled to the TEM. The size distribution or average size of the synthesized AgNPs were determined by dynamic light scattering (DLS) and zeta potential measurements were carried out using DLS (Malvern). For DLS analysis the samples were diluted 10 folds using 0.15M PBS (pH 7.4) and the measurements were taken in the range between 0.1 and 10,000 nm. X- Ray Diffraction (XRD) was done with the help of by X-Pert Pro Diffractometer. The X-ray diffraction data were obtained using step scan technique and with Cu-Ka radiation (1.500 Å, 40 kV, 30 mA) in h–2h configuration. The AgNPs were coated on to the glass substrate and after drying, the sample was analyzed by X-ray diffractometer. The crystallite domain size was calculated using the Debye–Scherrer’s formula. Finally, Fourier Transform Infra-red Spectroscopy (FTIR) was used for detection of different functional groups. The dried AgNPs were analyzed by ALPHA FT-IR Spectrometer (from Bruker, Germany) for the detection of different functional groups by showing peaks from the region of 4000 cm^-1^ to 500 cm^-1^.

### Multi drug resistant clinical isolates of *P. aeruginosa* from burn patients

Fifteen clinical isolates were obtained from the various samples of burn patients receiving in Microbiology Department of Pt. B.D.S. Post Graduate Institute of Medical Sciences, Rohtak, Haryana, India. The purity and identity of each isolate was confirmed in laboratory by standard microbiological methods [35-37]. The sources of the clinical isolates were urine, wounds, blood, and body fluids of burn patients. The approval of SRAC (Scientific & Research Advisory Committee) of the institute was taken for the study with reference no. UHS/OSD/2010/1 dated 27/02/2012. The 10 most cost-effective antibiotics routinely used to treat *P. aeruginosa* infections were employed in the susceptibility test. The antibiotics included were amikacin, aztreonam, ceftizoxime, cefepime, gentamicin, imipenem, netilmicin, ofloxacin, piperacillin and tazobactum. For isolation of MDR strains, these antibiotics were used and susceptibility was checked by Kirby-Bauer disc method [38]. The strain which were resistant to 6 or 7 antibiotics was taken as MDR strain.

### Antibacterial assay of AgNPs

4 Test samples of the AgNPs were prepared in DMSO (Dimethyl Sulfoxide). The concentrations of AgNPs were ranges from 12.5-100 μg/ml i.e. 12.5, 25, 50 and 100 μg/ml. The antimicrobial activities were determined by agar well diffusion assay [39]. Under aseptic conditions, in to the Bio safety chamber, 20 ml of MHA medium was dispensed in to pre-sterilized petridishes. Once the media solidifies it was then inoculated with micro-organism suspended in peptone water. The media was then punched with 6mm diameter hole and filled with different dilutions (varying from 12.5 to 100 μg/ml) of AgNPs extract from stock of 20 mg/ml. Streptomycin (10 μg/ml) was used as positive control and DMSO was used as a negative control. Finally, the petridishes were incubated for 24 hours at 37°C. The diameter of zone of inhibition was measured as indicated by clear area devoid of growth of microbes. Each experiment was done in triplicate.

Minimum inhibitory concentration method (MIC) was calculated by micro broth dilution method in 96 multi-well microtitre plates with slight modifications [40]. Qualitative experimentation was done by resazurin indicator solution prepared by dissolving a 270 mg tablet in 40 ml of sterile distilled water. Purple colour of indicator (resazurin) reduced in the presence of living bacteria. Colour change from purple to pink or to colourless. In the absence of living bacteria the colour of the indicator were remain purple. The lowest conc. at which colour change occurred was taken as MIC.

### Consent

Written informed consent was obtained from the patient for the publication of this report.

## Additional files

Additional file 1: Figure S1. Formation of silver nanoparticles of *P. amarus.* Figure showing colour change from pale yellow to dark brown. Additional file 2: Figure S2. Uv-Vis Spectra of silver nanoparticles of *P. amarus.* Figure showing Uv-Vis absorption spectra of silver nanoparticles at range of 300-800. Additional file 3: Figure S3. Picture of antibacterial activity of AgNPs against 15 MDR strains of *P aeruginosa.* Figure showing antibacterial activity of AgNPs against 15 MDR strains of *P aeruginosa* at concentration of 12.5-100 μg/ml.
