# Supplementary figures and images for: Green silver nanoparticles of Phyllanthus amarus: as an antibacterial agent against multi drug resistant clinical isolates of Pseudomonas aeruginosa
Source: J Nanobiotechnology. 2014 Oct 1;12:40. doi: 10.1186/s12951-014-0040-x (PMC4189661; doi:10.1186/s12951-014-0040-x)

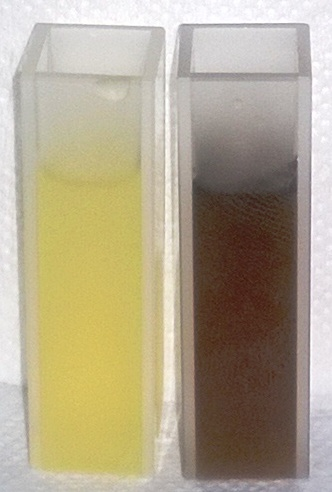

Supplement: Additional file 1: Figure S1. — Formation of silver nanoparticles of P. amarus. Figure showing colour change from pale yellow to dark brown. [file 12951_2014_40_MOESM1_ESM.tiff]

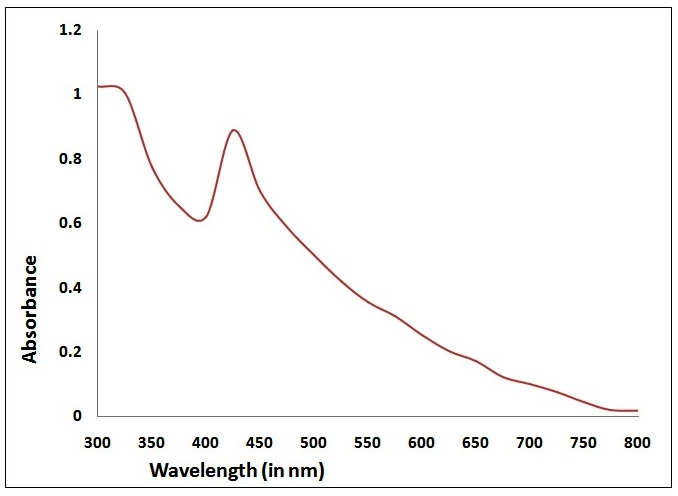

Supplement: Additional file 2: Figure S2. — Uv-Vis Spectra of silver nanoparticles of P. amarus. Figure showing Uv-Vis absorption spectra of silver nanoparticles at range of 300-800. [file 12951_2014_40_MOESM2_ESM.tiff]

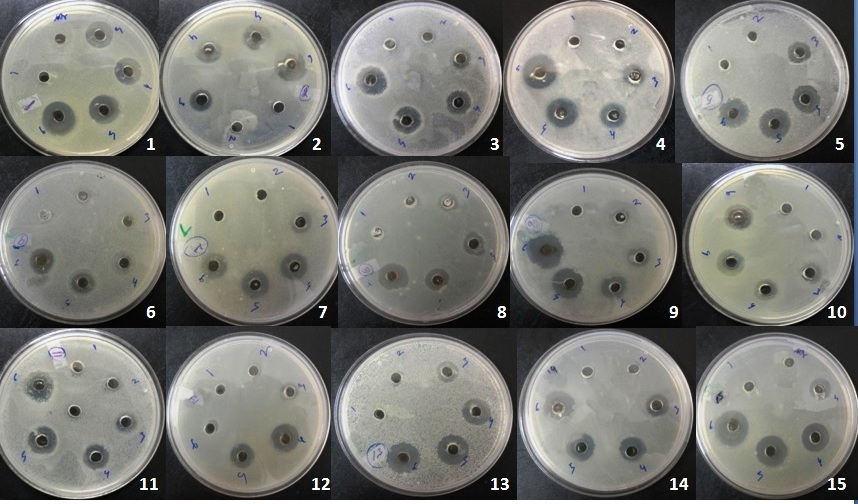

Supplement: Additional file 3: Figure S3. — Picture of antibacterial activity of AgNPs against 15 MDR strains of P aeruginosa. Figure showing antibacterial activity of AgNPs against 15 MDR strains of P aeruginosa at concentration of 12.5-100 μg/ml. [file 12951_2014_40_MOESM3_ESM.tiff]
